# Underdog or Top Dog Brand Story? The Role of Self-Construal and Need of Uniqueness

**DOI:** 10.3389/fpsyg.2021.765802

**Published:** 2021-11-26

**Authors:** Yalin Li, Min Zhao

**Affiliations:** ^1^School of Management, Suqian University, Suqian, China; ^2^School of Information and Communication Engineering, Hubei University of Economics, Wuhan, China

**Keywords:** self-construal, need for uniqueness, brand story, underdog, top dog

## Abstract

The design of an effective brand story has become a key issue in marketing strategies. This study aims to explore what kinds of brand stories (underdog or top dog) individuals prefer from the perspective of the level of self-construal and the need for uniqueness. In this study, a questionnaire survey was used to collect data from China. One-way analysis of variance and bootstrapping via the Process plug-in were adopted to test the hypotheses. This study confirms that individuals with independent self-construal have a higher need for uniqueness and prefer underdog brand stories, while individuals with interdependent self-construal have a lower need for uniqueness and prefer the top dog brand story. This paper promotes theoretical research in the fields of self-construal, the need for uniqueness, and brand stories, and provides rich theoretical support for enterprises in designing and adjusting brand stories. Implications, limitations and future studies are discussed.

## Introduction

Storytelling is an important and effective marketing tool (Kozinets, 2001; Hong et al., 2021) that enables people to dream and imagine a whole new world (Yueh and Zheng, 2019). It could be an effective marketing tool for branding (Gensler et al., 2013; Granitz and Forman, 2015; Solja et al., 2018; Kao, 2019).

Brand stories have a variety of themes and vary greatly in content, but the underdog story is one of the most popular, and the theme is prominent in collective fantasy and consciousness (Goldschmied et al., 2017). An underdog is a person or group who has a disadvantageous status in competition but makes arduous efforts (Paharia et al., 2011). The opposite of the underdog is the top dog, who is in an advantageous position in competition. Paharia et al. (2011) confirmed that the underdog brand story is more able to arouse consumer sympathy than the top dog brand story, thus positively affecting consumer response to the brand.

The difference between underdog and top dog brands is rooted in the market’s hierarchical structure and the strength differences between competitors (Jin and Huang, 2019). Some examples of underdog brands that appeal to their humble origins include Apple and Hewlett-Packard; other underdogs are currently embroiled in an unbalanced struggle (where big brands dominate smaller rivals) (Delgado-Ballester, 2020). Avis’s market campaigns based on the famous slogan “We’re No. 2. We try harder” prove the potential of the underdog position in the marketing environment (Jin and Huang, 2019). By clearly acknowledging its underdog position and concomitant enduring efforts, the company has achieved unprecedented success and turned a profit smoothly. The phenomenon that people root for the underdog by ignoring disadvantageous factors such as insufficient resources and little chance of success is called the underdog effect (Simon, 1954).

Although many theoretical studies and practical operations show that underdog brand stories are more acceptable to consumers, some scholars have also found that underdogs cannot be supported by consumers in any field, that some products or services are not suitable for the underdog brand story (McGinnis and Gentry, 2009; Li and Zhao, 2018) and that underdog brand positioning may not always be beneficial (Kim and Park, 2020). Kim et al. (2019) announced and proved the existence of an underdog trap, warning of the side effects of underdog positioning. Therefore, it is important for marketers to identify factors that affect the relative effectiveness of underdog positioning (Tang and Tsang, 2020). However, relatively few studies have been conducted in this area.

Research has shown when the information is consistent with personal motivation or personality traits (Hirsh et al., 2012), people will evaluate it more positively (e.g., regulatory fit; Florack and Scarabis, 2006). Each exhibits specific characteristics, attitudes, preferences and behavior depending on events (Popa et al., 2019). A fundamental and unexplored question with respect to preference of brand story is whether need for uniqueness and self-construal as personality characteristic have different preferences for brand stories? The need for uniqueness is a personality characteristic that reflects the extent to which consumers strive to establish individual uniqueness through consumption (Tian et al., 2001; Ruvio et al., 2008). The individual’s need for uniqueness shows those who are highly in need of uniqueness desire to be different from others and actively seek ways to meet their need for uniqueness (Lindsey-Hall et al., 2021). According to Markus and Kitayama (1991) self-construal describes how individuals perceive the relationship between the self and others. Individuals’ behavior patterns and their interactions can vary depending on their self-construals (Jebarajakirthy and Das, 2020). Therefore, this study examines how personality (need for uniqueness and self-construal) is linked to consumers’ preferences for brand stories.

Although previous researches have provided the basic understanding results about preferences for brand stories (underdog or top dog), several important and deeper issues have not yet been explored. First, previous studies on brand story preference mainly focused on the reasons why consumers choose the underdog brand story or the top dog brand story such as brand identification (Paharia et al., 2011). However, there are few studies on the boundary conditions of different brand stories. Second, in the past, the preference for brand stories mainly focused on perceived risk (e.g., Xu, 2014) and product types (e.g., Li and Zhao, 2018), and rarely focused on consumers’ personality characteristics. Therefore, the purpose of this study is to explore the influence of consumers’ personality characteristics (need for uniqueness and self-construal) on preference of different brand stories (underdog or top dog) and second, to analyze the effect of self-construal on customers’ need for uniqueness to clarify the relationship between need for uniqueness and self-construal. Finally, we examine whether the need for uniqueness plays a mediating role between self-construal and brand story preference to clarify the internal mechanism of personality characteristic (self-construal) affecting brand story preference.

We make some noteworthy contributions to the literature. First, to the best of our knowledge, this is the first study to examine the effect of self-construal on brand story preference, which will help managers better understand consumers’ behavior and develop more persuasive or cogent brand stories to target consumers. Second, this study contributes to uniqueness theory by revealing the relationship between the need for uniqueness and self-construal, which provides a new clue to explain the need for uniqueness. Third, this study provides a new theoretical guidance on how to better play the role of the underdog effect. This provides a new strategic guidance for how the disadvantaged party can give more support in the competition.

The rest of the paper is organized as follows. First, we discuss the literature review and present the research hypotheses. Next, we describe our methodology. The results of the study are then discussed. Finally, we conclude by discussing the theoretical and practical implications and future research directions.

## Literature Review

### Brand Story

Brand is an important variable in marketing and effective communication with different means can effectively enhance the brand image and promote purchase intentions (Dabija and Băbuţ, 2014; Bratu, 2019). In recent years, brand stories as a communication mean have emerged as significant marketing constructs (Huang, 2010; Woodside, 2010) and are widely used in marketing communication, which is defined as a means to convey the meaning of products and brands to customers (Lin and Chen, 2015). Elements such as origination, innovation and development, benefits and values, and visions can all be conveyed through a brand story (Lin and Chen, 2015). Storytelling is widely used as a customer engagement strategy (Van Laer et al., 2019).

Consumers can understand and perceive a brand through relevant associated stories (Escalas, 2004). Through these stories, consumers can understand the information and value of brands and products more clearly and quickly (Chiu et al., 2012). Storytelling has become an important component in persuading a broad audience (Manning and Bejarano, 2017). It has a positive impact on consumers’ brand emotions, attitudes, and behavioral intentions (Solja et al., 2018). One of the reasons storytelling can affect people is stories’ ability to evoke people’s emotions (Escalas, 2004) and make customers feel more engaged (Robiady et al., 2021), causing consumers to experience pleasure and happiness (Li et al., 2017; Lim and Childs, 2020). Moreover, storytelling inspires a sense of authenticity (Granata and Scozzese, 2019). Brand stories are more effective than other methods for transmitting advertising information. They not only improve consumers’ willingness to buy as well as their loyalty but also smoothly create brand differences and bring consumers closer to brands (Escalas, 2004; Rozier and Santos, 2011; Lundqvist et al., 2013). Therefore, controlling the power of a brand story is an effective way to establish a brand–consumer relationship (Lin and Chen, 2015) and build strong brands and brand loyalty (Lundqvist et al., 2013; Feng, 2018).

### Underdog and Top Dog Brand Stories

Brand stories can appeal to a variety of themes and values. Paharia et al. (2011) proposed four types of brand stories: underdog, privileged achiever, victim, and top dog. Underdogs are expected to lose the contest or struggle, as in sports or politics, and are at a disadvantage. The opposite of the underdog is the top dog, who is the favorite or the one expected to win in a competition. In the underdog brand story, the brand is described as being at an external disadvantage and lacking resources, but it has an indomitable spirit and maintains the enthusiasm and determination to pursue success through the brand story. In the top dog brand story, the brand is described as having abundant resources; it succeeds in the market by relying on its strong advantages. However, compared with the underdog brand story, its enthusiasm and determination are relatively low. Although the underdog is regarded as an undesirable and defeated party, it often arouses people’s recognition and support, and they expect that the underdog will be able to reverse the predicament and overcome the top dog (Paharia et al., 2011).

Traditionally, top dog brand stories have been considered favorable because individuals tend to associate themselves with winners and disconnect from losers (Saad, 2012). However, Paharia et al. (2011) proposed an opposite conclusion, which posited that underdog brand stories help increase purchase intentions, choice decisions, and brand loyalty. According to Paharia et al. (2011), the underdog brand story depicts an emerging trend in brand marketing, where enterprises narrate a historical review of their humble origins, scarce resources, and determined struggle against hardship. An underdog brand story comprises two fundamental elements: external disadvantage and passion and determination (Paharia et al., 2011). Enterprises are increasingly applying underdog brands as a marketing strategy, especially by utilizing underdog stories in brand advertising (Kao and Wu, 2019).

Being an underdog is extraordinary (He et al., 2020). Early research has shown that the underdog position can help small firms compete with large ones (Paharia et al., 2014). As such, many marketers choose to position their brand as an underdog in the market rather than as a top dog (Tang and Tsang, 2020). Many studies have investigated the positioning of underdog brands and their positive effects, confirming consumers’ motivation to support underdog brands (Paharia et al., 2011; Kao, 2015; MacInnis and Folkes, 2017; Goldschmied et al., 2018). Further study found that the characteristics of the target entity (importance of consequence and self-relevance) and consumers (underdog disposition, identification, and emotions) are the factors that foster the underdog effect (Shirai, 2017). Although underdog brand positioning has received considerable support, some scholars have pointed out that underdog brand positioning does not play a role at any time. For example, Kirmani et al. (2017) concluded that when consumers have a trade-off between brand quality and other brand attributes, underdog brand positioning will have a negative impact on purchase intention. Kim et al. (2008) also showed that when consumers’ physical or material interests are threatened, they will abandon the underdog and support the top dog. The boundary conditions (brand status, brand identification, firm characteristics, personal control, type of service providers or transgressions, product type, and psychological experience of power; e.g., Kao, 2015; Goldschmied et al., 2017; Kirmani et al., 2017; Berendt et al., 2018; Li and Zhao, 2018; Jin and Huang, 2019; Kim and Park, 2020; Tang and Tsang, 2020) of the underdog effect were also discussed.

### Need of Uniqueness

Some people will be more compliant with the opinions of others, abide by social norms, avoid criticism, gain recognition from others, and receive rewards for such behavior (Simonson and Nowlis, 2000), while others will be more individualistic. Some people refer to others’ opinions before making decisions. However, some people take anti-conformity actions to display their unique characteristics as markedly different from others (Simonson and Nowlis, 2000). Uniqueness is an individual-level characteristic (Fromkin and Snyder, 1977; Lynn and Harris, 1997; Tian et al., 2001) that is defined as distinguishing oneself from others by purchasing brands and products to enhance one’s own and social image (Tian et al., 2001), which emphasizes being distinct from others (Schumpe et al., 2016) and expresses a non-conformity tendency to look different from others (Snyder and Fromkin, 1977).

Individuals may think that being highly similar to others is unpleasant (Lynn and Harris, 1997) or may even view it as a threat to themselves (Snyder and Fromkin, 1977). To satisfy their desire for uniqueness, consumers obtain, use, and dispose of products that meet their desired personal and social identities (Tian and McKenzie, 2001). Research has shown that consumers’ purchasing decisions, consumption, and behaviors meet their need to maintain and promote their uniqueness (Lynn and Harris, 1997; Simonson and Nowlis, 2000; Cheema and Kaikati, 2010; Moldovan et al., 2015). People’s pursuit of uniqueness is not only for the purpose of identity but also to distinguish themselves from the excluded group in order to reduce the negative consequences of social exclusion (Bozkurt and Gligor, 2019).

Consumers’ need for uniqueness is an important variable that involves people using consumer goods to enhance their image by seeking characteristics that are different from those of others (Tian and McKenzie, 2001; Tian et al., 2001; Escalas and Bettman, 2003). Consumers with a high need for uniqueness pay more attention to self-expression, establish an independent identity, use distinctive brands (Shavitt, 1989), and make more risky decisions (Cantarella and Desrichard, 2020). Researchers have found that the need for uniqueness may also promote unconventional or even irrational decisions and behavior, such as a preference for more expensive products (Amaldoss and Jain, 2005) or liking an encounter that includes social discomfort (Sharifi, 2020). In this study, the need for uniqueness was used in the context of brand story preference.

### Self-Construal

Self-construal refers to individuals’ view of the relationship between themselves and others, which reflects their different views about themselves (Agrawal and Maheswaran, 2005) and affects whether individuals focus on themselves or on relations with others (Markus and Kitayama, 1991; Cross et al., 2002). Self-construal includes independent self-construal and interdependent self-construal (Markus and Kitayama, 1991). Independent individuals view themselves as independent entities that separate from other individuals, while interdependent individuals view themselves as related to others as part of a group (Li et al., 2019). The main characteristics of independent self-construal are that individuals are separated from others, paying attention to their own abilities, characteristics, and preferences, and that personal goals are prioritized ahead of those of in-groups (Fazeli et al., 2019). By contrast, people with interdependent self-construal view themselves as part of the social context in which they are connected with others, and they hold a self-explanation defined by their social associations (Kim and Johnson, 2014). They tend to value connectedness, conformity, and harmony (Jeon et al., 2020).

Although self-construal refers to the extent to which people perceive their relationship with others, researchers have found that these different views of self also affect various areas of consumers, such as price–quality relationships (Lalwani and Shavitt, 2013), brand extension evaluation (Ahluwalia, 2008). Some studies have also shown that self-construal affects thinking in general, guiding thoughts and interpretations related to others and objects (van Baaren et al., 2003).

## Hypothesis Development

### Self-Construal and Brand Story Preference

Self-construal refers to how people view their relationships with others and their social environment, which is divided into interdependent self-construal and independent self-construal (Markus and Kitayama, 1991). People with independent self-construal consider themselves to be autonomous and unique, and they focus on personal achievement. In contrast, people with interdependent self-construal think that they are connected to others and are part of their social context (Jeon et al., 2020). Individuals with independent self-construal tend to express their desires, preferences, attributes, and abilities, and their values and behavior patterns are not easily affected by others. On the contrary, individuals with interdependent self-construal tend to seek consistency with others, thus becoming part of a group, and their values or behavior patterns are easily influenced by others (Wang et al., 2017), which shows that independent self-construal individuals habitually pay more attention to themselves than other individuals, while interdependent self-construal individuals pay more attention to others than independent individuals (Wu et al., 2019).

Although many people sympathize with the underdog in many cultures, most people worship the top dog and aspire to be a top dog. Traditional research on social identity theory posits that people keep a distance from losers (underdog) (Tajfel and Turner, 1986). In market competition, consumers need to choose from among competitors. Driven by rationality, they often stand on the winner’s side (top dog) by selecting goods with better performance and higher popularity. Enterprises and marketers participating in the competition are usually unwilling to become an inferior party (underdog) (Zhong et al., 2014). Facing different brand stories, most people prefer to choose the top dog brand story. Aaker and Schmitt (2001) found that individuals with independent self-construal tend to express their differences from others, while individuals with interdependent self-construal tend to demonstrate similarity with their peers. Therefore, individuals with interdependent self-construal are more willing to choose the top dog brand story in order to maintain consistency with the majority of people. Based on the above analysis, this study proposes the following hypothesis:

H1: Compared with individuals with independent self-construal, individuals with interdependent self-construal prefer the top dog brand story.

### Self-Construal and Need for Uniqueness

Individuals with interdependent self-construal tend to emphasize relationships and connectedness, and they are more inclined to deal with and explain the relationships among objects. On the contrary, individuals with independent self-construal tend to focus on individuality and uniqueness, and therefore tend to focus on individual factors rather than the relationships among objects (Jeon et al., 2020). Prior research has indicated that people seek to differentiate themselves from other members in their group because they strive for uniqueness and differences (Snyder and Fromkin, 1977). When people’s identities are threatened because they are typified and considered to be highly similar to others, they tend to reduce the threat through counter-conformity (Tian et al., 2001).

Consumers with interdependent self-construal pay more attention to the product’s gregariousness, and consumers with independent self-construal intentions pay more attention to products’ uniqueness. Independent self-construal may influence consumers’ need for uniqueness. Based on the above analysis, this study proposes the following hypothesis:

H2: Compared with individuals with independent self-construal, individuals with interdependent self-construal have the lower need for uniqueness.

### Need for Uniqueness and Brand Story Preference

Need for uniqueness theory posits that the need to see oneself as unique is a potent and continuous force in our society (Snyder and Fromkin, 1977). For example, when people have a high need for uniqueness, they express their differences through observable behaviors, such as wearing logos or fashions that establish their differences (Workman and Kidd, 2000). A person’s need for uniqueness is the pursuit of distinguishing themselves from others in a particular social group and at the same time pointing out the social group to which they belong (Erasmus et al., 2016). Consumers will try to differentiate themselves from others more or less explicitly through their product choices (Erasmus et al., 2016).

When individuals are threatened by their own uniqueness, they regain self-esteem and reduce negative impacts through self-differentiated behavior (Snyder and Fromkin, 1977). Individuals may realize their desire to be unique in a variety of ways (Lee et al., 2017). For example, individuals can reflect their uniqueness by displaying their possessions, interpersonal interaction style, or knowledge and expertise in a certain field (Zhu et al., 2017). In addition, exclusive, rare, and unique brands are used to satisfy the need for uniqueness (Tian et al., 2001; Kauppinen-Räisänen et al., 2018).

Both theoretical and practical research have found that most people accept and choose the top dog. However, the need for uniqueness may be satisfied through unpopular choice behaviors, such as consuming products that deviate from social norms and carry the risk of social disapproval (Stiglbauer and Kovacs, 2019). Individuals with a high need for uniqueness may make different choices than most people in order to show their uniqueness, which may entail a stronger sense of identification with the underdog and support for the underdog brand story. On the contrary, individuals with a low need for uniqueness tend to make choices that are consistent with most people, thus supporting the top dog brand stories. Based on the above analysis, this study proposes the following hypothesis:

H3: Compared with individuals with high need for uniqueness, individuals with low need for uniqueness prefer the brand story of top dog brand story.

### Mediation Effect of Need for Uniqueness Between Self-Construal and Brand Story Preference

Individuals with different self-construal have different concerns regarding different judgments and decision-making. Specifically, individuals with independent self-construal tend to pay attention to the differences between themselves and things, while individuals with dependent self-construal are more inclined to focus on the similarities between themselves and things (Nisbett et al., 2001; Hong and Chang, 2015).

According to Triandis and Gelfand (1998), in the relationship between self and others, individuals with independent self-construal disregard competition with others in order to obtain a higher social status. Instead, they emphasize differences with other group members, focusing on pursuing uniqueness. Individuals with independent self-construal want to do as they like, but they do not insist on achieving a higher status through competition with other group members (Triandis and Gelfand, 1998; Gürhan-Canli and Maheswaran, 2000). Individuals with interdependent self-construal pay more attention to others and seek similarities or commonalities with other group members; while those with independent self-construal pay more attention to themselves, seek differences with others, and highlight their own uniqueness. Therefore, individuals with independent self-construal are more likely to accept an underdog brand story compared to individuals with dependent self-construal.

Choosing and using products is an important way to distinguish oneself from others and express uniqueness (Ruvio et al., 2008; Cheema and Kaikati, 2010). The purchase, use, and disposal of products can shape a person’s personal and social image and establish individual uniqueness through the pursuit of differences with others (Tian et al., 2001). Previous studies have shown that the level of consumer demand for uniqueness can positively affect consumers’ perception of product uniqueness (Song and Lee, 2013), and higher consumer demand for uniqueness can have a stronger sense of uniqueness for products with unique implications. Simonson and Nowlis (2000) found that consumers with a high demand for uniqueness are not only more eager to have unique products but are also more attracted to unique product designs. It can be inferred that if the brand story contains elements reflecting uniqueness, consumers with a high demand for uniqueness will show an obvious preference for it.

Compared with the top dog brand story, the underdog brand story is more distinctive and unique, so it can make people feel unique. Therefore, we can infer that consumers with a higher demand for uniqueness will show an obvious preference for underdog brand stories, while consumers with a lower demand for uniqueness will show a preference for the top dog brand story. In summary, this study infers that individuals with an independent self-concept will produce a stronger unique demand and form a preference for the underdog brand story. On the contrary, individuals with interdependent self-construal have a lower uniqueness requirement and tend to prefer the top dog brand story. Based on the above analysis, this study proposes the following hypothesis:

H4: Need for uniqueness plays a mediation role between self-construal and brand story preference.

**FIGURE 1:**
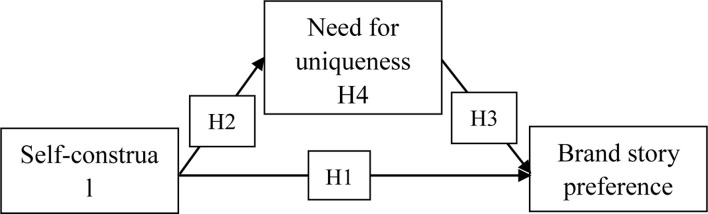
Theoretical model.

The theoretical model of this paper is shown in Figure 1.

## Methodology

### Data Collection

In this study, a questionnaire survey was used to collect data in a university in China. A total of 220 questionnaires were sent out, 10 questionnaires were rejected due to incomplete answers, and 210 valid questionnaires were finally included in the analysis. Participants’ basic information, including gender and age, was presented as demographic characteristics, where females accounted for 52.40%, males accounted for 47.60%, 53.30% were under 20 years old, 34.80% were between 21 and 25 years old, and 11.90% were over 25 years old.

#### Procedure

Referring to Li and Zhao (2018), participants were obtained using the central intercept test on a university campus in Wuhan, China. In the first step, participants were informed of the research purpose. In the second step, participants completed the self-construal and need for uniqueness scales. In the third step, participants judged their preferred brand story after reading the given brand stories. Finally, participants provided personal information.

#### Measures

This study involves three variables: self-construal, need for uniqueness, and brand story preference. To ensure content validity, these scales are mainly from published literature.

##### Need for Uniqueness

The measurement of need for uniqueness refers to the scale developed by Ruvio et al. (2008), the high cross-cultural validity of which has been proven through cross-cultural empirical research. The scale consists of 12 items, which include creative choice (4 items, such as “I often use mashup to create personally difficult to imitate”), non-popular choice (4 items, such as “I don’t follow the rules when buying products and choosing to use them”) and avoidance of similarity choice (4 items, such as “When the products I own become popular among ordinary people, I will reduce them for its frequency of use”). All items related to the variable were measured on a 7-point Likert scale (1 = *totally disagree*, 7 = *totally agree*).

##### Self-construal

The measurement of self-construal refers to the scale developed by Singelis (1994), which consists of 24 items and is divided into the independent self-construal and interdependent self-construal subscales. All items related to the variable were measured on a 7-point Likert scale (1 = *totally disagree*, 7 = *totally agree*).

##### Brand Story Preference

Brand story preference is measured by a single item, where “1” indicates liking the underdog brand story very much, “4” is neutrality, and “7” represents liking the top dog brand story very much.

##### Brand Story

According to Paharia et al. (2011), this study designs different brand story material, distinguished by two constructs: external resources and enthusiasm and determination. In the underdog brand story, information about the lack of external resources and higher enthusiasm and determination is emphasized. In the top dog brand story, information about rich external resources and lower enthusiasm and determination is emphasized.

#### Data Analysis Method

Data analysis in this study was performed using SPSS and Amos software, and the mediation effect test was completed via the bootstrapping method in the Process plug-in (Hayes, 2013), which is a multi-functional modeling tool that integrates the functions of existing and popular statistical tools for mediation and moderation analysis and can be used in SPSS for free (Sun et al., 2014). In this study, all bootstrapping analyses used 5,000 repeated samples to construct a confidence interval (CI) of 95% deviation correction. If zero is not included between the lower and upper limits of the CI, the corresponding effect is significant (Shrout and Bolger, 2002).

As this measurement model was developed based on *a priori* theory, which indicates how items are related to each of the target variables, confirmatory factor analysis (CFA) was adopted to test the measurement reliability and validity.

We then proceeded one-way analysis of variance (parametric) to test the significance the hypothesized relationships (H1, H2, and H3) in the proposed model. Finally, to test the mediation effect of need for uniqueness the bootstrapping analysis was conducted.

## Results

### Reliability and Validity

Cronbach’s α coefficient was used to evaluate the reliability of the scale. Data analysis showed that the Cronbach’s α coefficient of the need for uniqueness measurement scale was 0.973, that of the independent self-construal measurement scale was 0.964, and that of the interdependent self-construal measurement scale was 0.957. All the scales’ Cronbach’s α coefficients exceed the critical value of 0.7 (Nunnally, 1978). The scales used in this study had good reliability.

The measurement scale validity test mainly focused on item reliability and convergence validity. Item reliability mainly considers the factor load of each observation variable compared to its potential variable. In general, the standardized factor load should be greater than 0.70. Hair et al. (2010) also suggested that the square multiple correlation should be greater than 0.50 and reach a significant level (*P* < 0.05).

Convergence validity is mainly used to test the similarity of measurement results when different measurement methods are used to determine the same feature. The evaluation of convergence validity is based mainly on two criteria. First, the composite reliability (CR) of potential variables should exceed the critical value of 0.60 (Fornell and Larcker, 1981). The higher the CR value of the potential variables, the higher the CR value, indicating that these items measure the same potential variables. Second the average variance extracted should be higher than the critical value of 0.50 (Fornell and Larcker, 1981), which means that more than 50% variance can be explained by each factor extracted. The data analysis results showed that (see Tables 1–3), the relevant scale indicators used in this study exceeded the critical value and had good convergent validity.

**TABLE 1:** Reliability and convergent validity of need for uniqueness scale (*n* = 210).

**Construct**	**Indicator**	**Sig. test of parameters**				**Std.**	**Item reliability**	**Composite reliability**	**Convergence validity**	**Cronbach’s α**
					
		**Unstd.**	**S.E.**	**z-value**	**p**		**SMC**	**CR**	**AVE**	
need for uniqueness(M = 3.716,SD = 1.367)	cc1	1.000				0.782	0.612	0.974	0.755	0.973
	cc2	1.212	0.081	14.936	***	0.886	0.785			
	cc3	1.109	0.077	14.324	***	0.859	0.738			
	cc4	1.304	0.086	15.121	***	0.894	0.799			
	npc1	1.000				0.875	0.766			
	npc2	1.004	0.056	18.078	***	0.873	0.762			
	npc3	1.018	0.058	17.472	***	0.859	0.738			
	npc4	1.048	0.057	18.292	***	0.878	0.771			
	asc1	1.000				0.899	0.808			
	asc2	1.037	0.053	19.616	***	0.883	0.780			
	asc3	0.966	0.050	19.399	***	0.879	0.773			
	asc4	0.943	0.052	18.047	***	0.852	0.726			

***p < 0.001; χ^2^ = 65.689, df. = 51, χ^2^/df = 1.288(< 3), *p* = 0.081, RMR = 0.036(< 0.05), GFI = 0.951(> 0.90), NFI = 0.977(> 0.90), CFI = 0.995(> 0.90); RMSEA = 0.037(< 0.08). AVE, Average Variance Extracted; SMC, Square Multiple Correlation; CR, Composite Reliability.

**TABLE 2:** Reliability and convergent validity of interdependent self-construal scale (*n* = 210).

**Construct**	**Indicator**	**Sig. test of parameters**				**Std.**	**Item reliability**	**Composite reliability**	**Convergence validity**	**Cronbach’s α**
					
		**Unstd.**	**S.E.**	**z-value**	**p**		**SMC**	**CR**	**AVE**	
interdependent self-construal(M = 4.183,SD = 0.928)	sc1	1.000				0.798	0.637	0.958	0.657	0.957
	sc3	1.099	0.090	12.281	***	0.755	0.570			
	sc4	1.030	0.069	14.859	***	0.867	0.752			
	sc5	1.083	0.083	13.034	***	0.790	0.624			
	sc8	0.982	0.072	13.549	***	0.813	0.661			
	sc9	0.942	0.074	12.749	***	0.777	0.604			
	sc12	1.231	0.086	14.377	***	0.848	0.719			
	sc13	1.101	0.084	13.037	***	0.790	0.624			
	sc14	1.067	0.079	13.523	***	0.811	0.658			
	sc15	1.087	0.079	13.791	***	0.823	0.677			
	sc20	0.933	0.072	12.915	***	0.785	0.616			
	sc24	1.049	0.072	14.622	***	0.858	0.736			

***p < 0.001; χ^2^ = 93.751, df. = 54, χ^2^/df = 1.736(< 3), *p* = 0.001, RMR = 0.034(< 0.05), GFI = 0.929(> 0.90), NFI = 0.956(> 0.90), CFI = 0.981(> 0.90); RMSEA = 0.059(< 0.08). AVE, Average Variance Extracted; SMC, Square Multiple Correlation; CR, Composite Reliability.

**TABLE 3:** Reliability and convergent validity of independent self-construal scale (*n* = 210).

**Construct**	**Indicator**	**Sig. test of parameters**				**Std.**	**Item reliability**	**Composite reliability**	**Convergence validity**	**Cronbach’s α**
					
		**Unstd.**	**S.E.**	**z-value**	**p**		**SMC**	**CR**	**AVE**	
independent self-construal (M = 4.468,SD = 0.958)	sc2	1.000				0.893	0.797	0.964	0.691	0.964
	sc6	0.836	0.049	17.207	***	0.839	0.704			
	sc7	0.936	0.051	18.272	***	0.863	0.745			
	sc10	1.017	0.059	17.317	***	0.842	0.709			
	sc11	0.917	0.057	16.161	***	0.813	0.661			
	sc16	0.916	0.057	16.080	***	0.811	0.658			
	sc17	0.943	0.053	17.909	***	0.855	0.731			
	sc18	0.859	0.058	14.914	***	0.779	0.607			
	sc19	0.930	0.050	18.577	***	0.869	0.755			
	sc21	0.890	0.060	14.807	***	0.776	0.602			
	sc22	0.904	0.051	17.695	***	0.850	0.723			
	sc23	0.868	0.058	14.871	***	0.778	0.605			

***p < 0.001; χ^2^ = 121.976, df. = 54, χ^2^/df = 2.259(< 3), *p* < 0.05, RMR = 0.034(< 0.05), GFI = 0.913(> 0.90), NFI = 0.949(> 0.90), CFI = 0.971(> 0.90); RMSEA = 0.078(< 0.08). AVE, Average Variance Extracted; SMC, Square Multiple Correlation; CR, Composite Reliability.

### Common Method Deviation

In view of possible common method deviation of the data, this study uses the Harman single factor method for testing. Factor analysis showed that the first common factor’s variance interpretation percentage was 23.392%, which is less than 40% of the critical value (Podsakoff et al., 2003). There were no common method deviations in this study.

### Hypothesis Testing

According to Singelis’s method (1994), 42 subjects with absolute value of the difference between their independent self-construal score and their interdependent self-construal score that was less than or equal to 0.2 were excluded. The subjects whose difference between their independent self-construal score and their interdependent self-construal score was greater than 0.2 are regarded as having independent self-construal (M = 1.641, SD = 1.117, *n* = 102), and subjects whose difference between their independent self-construal score and their interdependent self-construal score was less than –0.2 are regarded as having interdependent self-construal (M = −1.645, SD = 1.087, *n* = 66).

One-way analysis of variance showed that there was a significant difference in individual brand story preference among individuals with different self-construal (F(1,167) = 703.697, *P* < 0.05, η2 = 0.81). Specifically, individuals with independent self-construal prefer the underdog brand story, while the individual with interdependent self-construal prefers the top dog brand story (M _independent self–construal–brand story preference_ = 2.147, SD_independent self–construal–brand story preference_ = 0.998 vs. M_interdependent self–construal–brand story preference_ = 6.046, SD _interdependent self–construal–brand story preference_ = 0.812). H1 was confirmed in the present study.

To verify H2, a one-way analysis of variance was used in this study. The data analysis results showed that there was a significant difference in individual need for uniqueness among persons with different self-construal (F(1,167) = 734.438, *P* < 0.05, η2 = 0.81). Furthermore, individuals with independent self-construal have a significantly higher need for uniqueness than individuals with interdependent self-construal (M _independent self–construal–need for uniqueness_ = 4.990, SD _independent self–construal–need for uniqueness_ = 0.765 vs. M _interdependent self–construal–need for uniqueness_ = 2.346, SD _interdependent self–construal–need for uniqueness_ = 0.256). Thus, H2 was supported.

Taking brand story preference as the dependent variable and need for uniqueness as the independent variable, a regression analysis was carried out. The results of the data analysis showed that need for uniqueness has a significant impact on individual brand story preference (F(1,167) = 2003.420, *P* < 0.05; b = −1.422, t = –44.760, *P* < 0.05). That is, the lower the need for uniqueness, the greater the preference for the top dog brand story. Thus, H3 was confirmed.

In this study, the mediation effect was tested via bootstrapping analysis using the Process plug-in (Model 4). To judge the mediation effect, this study adopted Shrout and Bolger’s (2002) mainstream view. The results of the data analysis showed that individuals’ self-construal has a significant impact on brand story preference through the need for uniqueness, for which the mediation effect coefficient is 3.155, with a 95% CI [2.703, 3.729]. Given that the CI of this effect does not contain zero, the mediation effect of need for uniqueness is significant, and H4 was supported.

## Conclusion

### Findings

Many studies have verified the underdog effect (Avery et al., 2010; Paharia et al., 2011). In a social psychology and political communication study, some scholars supported the role of underdogs (Vandello et al., 2007; Goldschmied and Vandello, 2009). However, the underdog effect is not unconditional, and few studies have explored its range of applications. This paper discusses need for uniqueness and self-construal in order to improve the effectiveness of underdog brand stories.

This study shows that different types of self-construal have a significant impact on brand story preference (F(1,167) = 703.697, *P* < 0.05; M _independent self–construal–brand story preference_ = 2.147 vs. M_interdependent self–construal–brand story preference_ = 6.046), and need for uniqueness plays a mediating role in this relationship (95% CI [2.703, 3.729]). Specifically, individuals with independent self-construal prefer the underdog brand story, while the individual with interdependent self-construal prefers the top dog brand story. In other words, the underdog effect can play a more important role to the individuals with independent self-construal. The reason why self-construal has an impact on brand story preference (underdog or top dog) is mainly related to personal need for uniqueness. Namely, individuals show their need for uniqueness with the help of brand story preferences. The higher the need for uniqueness, the greater the preference for the underdog brand story. Moreover, individuals with independent self-construal have a significantly higher need for uniqueness than individuals with interdependent self-construal. Speaking simply, individuals with independent self-construal have a higher need for uniqueness, and in order to maintain this out of the ordinary, they prefer the underdog brand story.

### Theoretical Implications

Previous studies on the affecting factors of brand stories preference mainly focused on perceived risk (Xu, 2014), product type (Li and Zhao, 2018) and cultural differences (Zhong et al., 2014), and rarely paid attention to the influence of personal characteristics. This paper chooses two personality traits: self-construal and need for uniqueness as the research perspective to analyze the impact of these personality traits on brand story preference. This study verifies the influence of different types of self-construal on brand story preference, which enriches underdog effect theory. Previous studies have confirmed the existence of the underdog effect, but the effect does not play a role in any situation. The current research puts forward a new theory from the perspective of self-construal.

This study confirms the mediating role of the need for uniqueness between self-construal and brand story preference. Based on Triandis and Gelfand’s (1998) research, this study reveals that an individual’s need for uniqueness in dealing with others is that the individual does not always show opposition or conflict with others; rather, such an individual is different from others, which seems to be an alternative to the group. Individuals with different self-construal show different levels of need for uniqueness, which, in turn, influences their preference for brand stories.

### Managerial Implications

The current research shows that the level of self-construal has a significant impact on brand story preference. Therefore, the use of relevant information to induce different types of self-construal in marketing communication will help cultivate consumers’ preferences for specific brand stories. For example, when an enterprise designs an underdog brand story, the narrative can convey information about “I” and “myself” that advocates independent self-construal or induces consumers to associate with their own unique scenes (such as “different me,” “special me,” etc.). On the contrary, when enterprises highlight the top dog brand story, they can use “we” or “our” and other information related to interdependent self-construal in marketing communication, inducing consumers to associate with the similarities between themselves and the group, so as to effectively enhance consumers’ acceptance or preference for a different brand story.

The current study confirms that need for uniqueness has an important impact on brand story preference. Therefore, enterprises can design or adjust brand stories according to consumers’ need for the uniqueness. According to the situation, adding some elements of a brand story flexibly can not only activate the brand but also convey the brand’s theme in a specific period. It is evident that this paper’s conclusion is an important reference for enterprises’ brand strategies.

### Limitations and Future Research

This study designs brand stories using Paharia et al.’s (2011) underdog brand dimension, which involves no modified sentences or more complex sentence structures. In the future, we will refer to other brand stories for design research.

Current studies use scales to measure individual self-construal. Although this method has been used in many studies, there are some shortcomings in self-report data collection. In the future, experimental priming methods can be used to stimulate individuals’ different levels of self-construal in order to enrich the existing research conclusions. In particular, with the continuous progress of technology, brain-based methods or neuroscientific approaches (Drugău-Constantin, 2019), which have an advantage over the established ones of marketing analyses to grasp and delve into the conduct of consumers (Mirică (Dumitrescu), 2019) are attracting the attention of theoretical and practical circles. In the future, this method can be used to study consumer behavior.

## Data Availability Statement

The raw data supporting the conclusions of this article will be made available by the authors, without undue reservation.

## Ethics Statement

Ethical review and approval was not required for the study on human participants in accordance with the local legislation and institutional requirements. Written informed consent for participation was not required for this study in accordance with the national legislation and the institutional requirements.

## Author Contributions

YL designed and drafted the manuscript. MZ collected and analyzed the data. Both authors contributed to the manuscript and approved the submitted version.

## Conflict of Interest

The authors declare that the research was conducted in the absence of any commercial or financial relationships that could be construed as a potential conflict of interest.

## Publisher’s Note

All claims expressed in this article are solely those of the authors and do not necessarily represent those of their affiliated organizations, or those of the publisher, the editors and the reviewers. Any product that may be evaluated in this article, or claim that may be made by its manufacturer, is not guaranteed or endorsed by the publisher.
